# Local Effects of Steroid Hormones within the Bone Microenvironment

**DOI:** 10.3390/ijms242417482

**Published:** 2023-12-14

**Authors:** Luca F. Sandor, Reka Ragacs, David S. Gyori

**Affiliations:** Department of Physiology, School of Medicine, Semmelweis University, 1085 Budapest, Hungary

**Keywords:** extraglandular steroidogenesis, de novo steroidogenesis, steroid hormones, bone metabolism, osteoclast, osteoblast, osteocyte, bone tumors, bone metastasis, pregnenolone derivates

## Abstract

Steroid hormone production via the adrenal cortex, gonads, and placenta (so-called glandular steroidogenesis) is responsible for the endocrine control of the body’s homeostasis and is organized by a feedback regulatory mechanism based on the hypothalamus–pituitary–steroidogenic gland axis. On the other hand, recently discovered extraglandular steroidogenesis occurring locally in different tissues is instead linked to paracrine or autocrine signaling, and it is independent of the control by the hypothalamus and pituitary glands. Bone cells, such as bone-forming osteoblasts, osteoblast-derived osteocytes, and bone-resorbing osteoclasts, respond to steroid hormones produced by both glandular and extraglandular steroidogenesis. Recently, new techniques to identify steroid hormones, as well as synthetic steroids and steroidogenesis inhibitors, have been introduced, which greatly empowered steroid hormone research. Based on recent literature and new advances in the field, here we review the local role of steroid hormones in regulating bone homeostasis and skeletal lesion formation. The novel idea of extraglandular steroidogenesis occurring within the skeletal system raises the possibility of the development of new therapies for the treatment of bone diseases.

## 1. Introduction

Steroid hormones exhibit diverse effects on regulating cell metabolism, development, reproduction, and salt–water balance, as well as on the functions of the immune, nervous, and skeletal systems [1]. The synthesis of steroid hormones often starts from cholesterol, which is also known as de novo steroidogenesis [1]. During this process, cholesterol is transported from the cytoplasm to the mitochondria [2]. Within the mitochondria, a key enzyme, Cyp11a1, is localized, which catalyzes the conversion of cholesterol to other steroid hormones [2]. Cyp11a1 is also termed a P450 side chain cleavage enzyme, and it is responsible for the first and enzymatically rate-limiting step of de novo steroidogenesis [3], while the steroidogenic acute regulatory protein (StAR) facilitates the transport of cholesterol into the mitochondria [4]. The movement of cholesterol across the mitochondrial membrane by the StAR protein is considered to be the rate-limiting step of acute steroid hormone synthesis [4]. The product of Cyp11a1, pregnenolone, is the precursor of all other steroids [3].

Further conversion of the steroid hormones is mediated by two major classes of enzymes, namely the cytochrome P450 (Cyp) and hydroxysteroid dehydrogenase (Hsd) enzyme families [5,6]. They are located either within the mitochondria or in the cytoplasm of hormone-producing cells; and electron donor molecules, such as NADH/NAD+ or NADPH/NADP+, are critical cofactors for their function [5,6]. The cellular and molecular mechanisms of de novo steroidogenesis have been extensively studied in glandular organs, e.g., the adrenal cortex, gonads, and placenta.

## 2. Glandular Steroidogenesis

The adrenal cortex has three layers, and its zona glomerulosa is responsible for the synthesis of mineralocorticoids (e.g., aldosterone), zona fasciculata for the production of glucocorticoids (e.g., cortisol), and zona reticularis for the secretion of the precursors of sexual steroids (e.g., dehydroepiandrosterone (DHEA) and androstenedione (A4)) [7,8,9,10,11,12]. Mineralocorticoids control salt–water balance, while glucocorticoids regulate cellular metabolism and key functions of both the innate and adaptive immune systems.

Steroidogenesis in the gonads is important for the production of androgens, estrogens, and progestogens. Gonadal steroidogenesis in the testis occurs within the Leydig cells, and it is responsible for the production of androgens [13]. Steroidogenesis in the ovary takes place in the theca interna and granulosa cells and results in the production of estrogens [14]. Additionally, the corpus luteum plays an important role in the production of progestogens [15]. Androgens (e.g., testosterone and dihydrotestosterone), estrogens (e.g., estrone, estradiol, and estrone), and progestogens (e.g., progesterone) regulate reproductive functions. Estrogens and progestogens are also synthesized by the placenta in pregnant women [16].

The hypothalamic–pituitary axis regulates the production of steroid hormones via the adrenal cortex, gonads, and placenta. Briefly, the hypothalamus produces releasing hormones, like CRH and GnRH, which stimulate the secretion of adenohypophyseal or trophormones via the anterior lobe of the pituitary [1,2]. In turn, the trophormones, such as ACTH, FSH, and LH, stimulate the steroidogenic gland to produce steroid hormones, which complete the system by having a negative feedback effect on the hypothalamus and pituitary, by inhibiting further stimulation of peripheral glands [1,2]. Estrogens can also exert positive feedback on the hypothalamus–pituitary axis during LH surge before the ovulation in the ovarian cycle [14]. 

**Figure 1:**
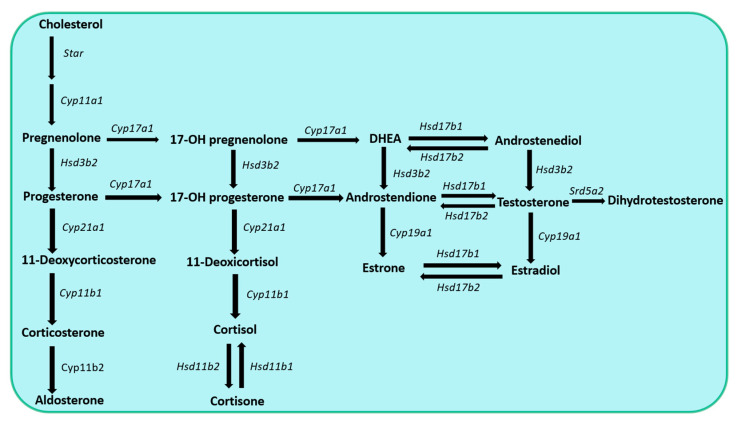
Overview of de novo steroidogenesis in the adrenal cortex and gonads.

Pregnenolone is the precursor for all steroid hormones, and the synthetic pathways for these steroid hormones are described in detail in Figure 1. Recently, pregnenolone and its derivates have been discovered to play locally important roles during the process of extraglandular steroidogenesis within different other tissues, including the skin, the adipose tissue, the intestine, as well as the nervous and immune systems [17,18].

## 3. Extraglandular Steroidogenesis

**Figure 2:**
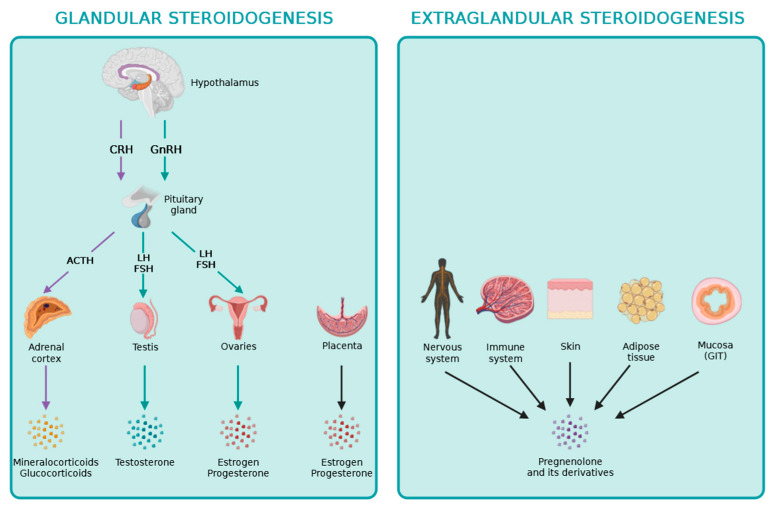
Overview of glandular and extraglandular steroidogenesis in humans.

De novo steroidogenesis taking place in other organs besides the adrenals, gonads, and placenta is termed extraglandular steroidogenesis [18]. The role of extraglandular steroidogenesis in the nervous and immune systems, skin, adipose tissue, and intestine has been reported previously, as it is indicated in Figure 2. The identification of local extraglandular steroidogenesis has been recently revitalizing the field.

### 3.1. Local Steroidogenesis within the Thymus

Groundbreaking work by Ashwell and his colleagues gave us the first evidence for extraglandular steroidogenesis taking place in the thymus and shed light on a novel area of steroid hormone research [19]. De novo glucocorticoid synthesis occurring in thymic epithelial cells was shown to be important for antigen-specific T cell selection by providing a survival cue against cell death induced by too strong TCR activation during negative selection, allowing positively selected thymocytes to survive [19]. Furthermore, inhibition of thymic corticosterone production was described to enhance TCR activation-induced apoptosis and enhanced negative selection of T cells [19]. Besides thymic epithelial cells, mature T cells are also capable of synthesizing glucocorticoids, which will be discussed in detail later as well.

### 3.2. Local Steroidogenesis within the Nervous System

Next, evidence indicated that locally produced steroids, so-called neurosteroids, could play important roles in the nervous system. In these studies, cholesterol-transporting StAR protein was found to be expressed within neurons and glial cells in both mouse and human brains [20]. Moreover, glial StAR co-localized with Cyp11a1 and it proved to be inducible with forskolin or dibutyryl cAMP [20]. De novo synthesized pregnenolone and its derivates were able to modify neuronal activity in further experiments by modulating GABA_A_ receptor function and had analgesic, sedative, and anesthetic properties in the experimental animals [21].

### 3.3. Local Steroidogenesis within the Immune System

According to the latest data, besides thymic epithelial cells, some immune cells are also capable of synthesizing and metabolizing steroid hormones [22]. Synovial macrophages were described to express functional androgen receptors in both males and females as well, and they were capable of metabolizing testosterone to active dihydrotestosterone [23]. In another study, human alveolar macrophages were shown to convert androstenedione to androgens, which could regulate their phagocytic activity [24]. Mouse macrophages were also detected to be able to produce androstenedione, testosterone, and estrogens depending on the influence of local factors, e.g., lipopolysaccharide (LPS) [25].

Furthermore, accumulating evidence indicated the effect of steroid hormones on NK cells [26,27,28,29,30,31,32,33,34,35]. Krukowski and her colleagues described that the release of glucocorticoids leads to the suppression of NK cell activity and the alteration of their cytokine production [26]. Glucocorticoids regulate NK cell function at least in part via epigenetic mechanisms, e.g., by decreasing the accessibility of promoters of interferon-gamma (IFNγ), perforins, and granzyme B [26,27,28]. In another study, the administration of exogenous glucocorticoids also decreased the surface expression of NK cell-activating receptors NKp30 and NKp46 [29]. Importantly, endogenous glucocorticoids upregulate checkpoint receptor PD-1 expression on NK cells during pathologic conditions and in disease progression [30,31]. It was also demonstrated that NK cells express estrogen receptors and can respond to estrogens [32]. Surprisingly, estradiol regulates NK cell activity via estrogen receptor (ER) beta, but not ERα [32]. Furthermore, NK cells play important roles during pregnancy, where estrogens and progesterone increase the expression of integrins and selectins [33,34], as well as chemokine receptors, on NK cells [35].

Dendritic cells were also indicated to be regulated by steroid hormones [36]. Glucocorticoids induce a tolerogenic phenotype in dendritic cells [37] by both controlling the maturation [38,39] and apoptosis of dendritic cells [40,41]. Furthermore, exogenous glucocorticoids inhibit antigen uptake and processing via dendritic cells [42,43]. In contrast, endogenous glucocorticoids suppress dendritic cell-derived cytokine secretion [44]. On the other hand, estrogens promote the differentiation of dendritic cells from bone marrow precursors [45], while progesterone during pregnancy reverts the effects of estrogen, leading to a more tolerogenic dendritic cell phenotype [46].

Steroid hormones influence both the development and function of T and B cells. According to the latest data, endogenous glucocorticoids enhance interleukin-7 receptor signaling in T lymphocytes during their development [47]. Importantly, exogenous glucocorticoids suppress both Th_1_ [48] and Th_2_ responses [49] via regulation of key transcription factors, T-bet and GATA3, respectively. Glucocorticoids also inhibit CD8^+^ cytotoxic T lymphocytes by upregulating the expression of inhibitory receptors, e.g., PD-1 [50]. Furthermore, glucocorticoids induce the development and enhance the function of immunosuppressive regulatory T (Treg) cells [51,52,53,54]. In pregnant women, glucocorticoids, together with estrogens, increased Treg numbers in peripheral blood [54]. Testosterone and estrogens regulate the function of T and B lymphocytes [55]. Estrogens enhance INFγ secretion by Th_1_ cells [56] but inhibit pro-inflammatory Th_17_ [57] and cytotoxic CD8^+^ T cells [58]. Glucocorticoids block B cell development by inducing apoptosis of B cells [59], while testosterone and estrogens have an opposite effect on B cell maturation [60,61].

The above-mentioned data indicated the ability of immune cells to respond and convert steroids. However, immune cells can also synthesize steroid hormones according to the most recent results. So-called de novo steroidogenesis was described in Th_2_ cells within the tumor microenvironment [62]. In these studies, immune cell-mediated steroidogenesis was proposed to elicit local immunosuppression via the inhibition of anti-tumorigenic immune cell subsets and promoted solid tumor growth [63]. The authors implicated pregnenolone as a “lymphosteroid” produced by Th_2_ lymphocytes. They also speculated that this de novo steroidogenesis might be an intrinsic characteristic of Th_2_ responses hijacked by cancer cells to actively induce tumor immunosuppression [62,63].

### 3.4. Local Steroidogenesis within the Skin

Hannen and her colleagues demonstrated that primary human keratinocytes could metabolize pregnenolone to cortisol [64]. They also showed that the epidermis and keratinocytes express all the enzymes required for cortisol synthesis, including Cyp11a1, Cyp17a1, Hsd3b2, Cyp21, and Cyp11b1 [64]. Furthermore, they showed that human skin expresses cholesterol transporter StAR [64]. The expression of StAR was found to be aberrant in skin disorders, including psoriasis and atopic dermatitis, suggesting dysregulation of steroid hormone synthesis in patients as well [65].

### 3.5. Local Steroidogenesis within the Adipose Tissue

Adipose tissue is one of the largest endocrine tissues of the body and it was shown to be an active site for steroid hormone metabolism and storage. According to recent data, steroid hormone precursors delivered to adipose tissue are further converted locally to regulate tissue metabolism and systemic steroid hormone levels [66,67]. Moreover, it was demonstrated that adipose tissue could express the enzymatic machinery for de novo steroidogenesis [66]. A study by Byeon and Lee provided evidence for the expression of key steroidogenic enzymes, including Hsd3b2, Cyp17a1, Cyp17b1, and Cyp19, both in male and female rat adipose tissues [66]. Local production of steroid hormones by adipocytes derived from mouse 3T3-L1 cells was also reported in another paper [67]. These studies suggest that adipose tissue is not only a target of steroids but can also de novo synthetize steroid hormones.

### 3.6. Local Steroidogenesis within the Intestinal Mucosa

Finally, accumulating evidence indicated the role of steroid hormone synthesis within the intestinal epithelium in the regulation of immune homeostasis as well as in the development of intestinal tumors and inflammatory bowel disease. In a study by Cima et al., the authors reported that epithelial cells of the intestinal mucosa express steroidogenic enzymes and release glucocorticoids, e.g., corticosterone, in response to T cell activation [68]. Intestinal mucosa-derived corticosterone exhibited an inhibitory role on T cells, and in the absence of mucosal glucocorticoids, enhanced T cell activation was observed in a mouse model of gastrointestinal viral infection [69]. Furthermore, liver receptor homolog 1 (Lrh1), which transcriptionally regulates the expression of steroidogenic enzymes such as Cyp11a1, Cyp17, Hsd3b2, and Cyp11b1, was implicated in the development of colon cancer [70]. Reduced expression of Lrh1 was observed in colon biopsies from patients with inflammatory bowel disease [71].

## 4. Local Effects of Steroid Hormones on Bone Cells

Steroid hormones play an important role in the bone microenvironment. Glucocorticoids and sexual steroids can regulate the cellular composition of bone tissue. They exert a direct effect on bone-forming osteoblasts, while the actions of steroid hormones on bone-resorbing osteoclasts are rather indirect and mediated by osteoblasts. Additionally, they influence the survival of osteocytes, and this way, they alter the tight coupling between bone formation and resorption, also known as bone remodeling. Importantly, both glandular and extraglandular steroidogenesis have an impact on bone cells and can regulate skeletal homeostasis, according to recent evidence.

Sexual steroids play an important role in the development and maintenance of the skeletal system. Androgens can stimulate periosteal bone growth in both genders [72]. Since men have higher androgen hormone levels, they reach higher cortical thickness and peak bone mass. It was believed in the past that estrogens regulate bone homeostasis in women, while testosterone is responsible for it in men. However, a landmark study showed that in men treated with gonadotropin blockade and aromatase inhibition, estrogens account for at least 70% of the effect of sex steroid hormones on bone metabolism [73]. Further studies pointed out that estrogens more strongly regulate bone mass than androgens [72]. However, there are differences in the site-specific actions of sex hormones. In men, estrogens control cortical bone homeostasis, while testosterone regulates trabecular bone turnover [72]. The most significant effect of glucocorticoids is an inhibition of bone formation and enhancement of bone resorption. On the cellular level, all steroid hormones regulate osteoblasts, osteocytes, and osteoclasts, which will be discussed in detail next.

### 4.1. Role of Steroid Hormones on Osteoblasts

Osteoblasts are terminally differentiated mononuclear cells of mesenchymal origin that can synthesize both the organic and inorganic phases of the bone. They are derived from pluripotent mesenchymal stem cells, which can also differentiate into adipocytes, chondrocytes, and fibroblasts. Osteoblast development is critically dependent on the Wnt/β-catenin and bone morphogenetic protein (Bmp) signaling pathways [74]. The formation of new bone is mediated by osteoblasts via the release of structural proteins (e.g., type I collagen, osteonectin, and osteocalcin) and by driving the deposition of hydroxyapatite crystals via the expression of alkaline phosphatase [74]. Developing osteoblasts express type I collagen, osteonectin, osteocalcin, and alkaline phosphatase, while mature osteoblasts undergo apoptosis or become osteocytes.

#### 4.1.1. Effects of Glucocorticoids on Osteoblasts

Osteoblasts are the main cellular targets of glucocorticoids in the skeletal system. Glucocorticoids decrease the number of osteoblasts and their function. Exposure to supraphysiological levels of glucocorticoids restricts osteoblastogenesis, and glucocorticoid excess shortens the lifespan of mature osteoblasts by promoting their apoptosis [75]. Furthermore, high levels of cortisol can diverge stromal progenitor cell development toward adipogenesis [75]. Glucocorticoids regulate the survival and lifespan of osteoblasts by endocrine as well as autocrine and paracrine effects [75]. Glucocorticoids decrease osteoblast function via the modulation of growth factor receptor expression, signaling, and binding. Glucocorticoids were shown to inhibit the secretion of Wnt (e.g., Wnt7b, Wnt10, and Wnt16) and Bmp proteins (e.g., Bmp2) [76,77,78]. Moreover, suppression of growth factors (e.g., insulin-like growth factor I (Igf-I)) and cytokines (e.g., interleukin-11) also contribute to the inhibitory effects of glucocorticoids on osteoblasts [79,80,81].

#### 4.1.2. Effects of Sex Steroids on Osteoblasts

Estrogens and androgens are important regulators of bone metabolism [73]. Estrogen acts via two receptors, namely estrogen receptor-alpha (ERα) and estrogen receptor-beta (ERβ) [82]. ERα in osteoblasts was found to be responsible for the majority of protective effects of estrogens and promote cortical bone accrual in response to mechanical stimulus [83]. A high number of estrogen-induced genes in osteoblasts was described [84]. Estrogens increase the expression of alkaline phosphatase and type I collagen as well as modulate the responsiveness of their receptors for 1,25(OH)_2_D_3_ and PTH in osteoblastic cells [85]. Estrogens were also demonstrated to inhibit osteoblast apoptosis and increase osteoblast lifespan [84]. At the molecular level, interference with MAPK pathways and transcription factors such as c-Jun and c-Fos mediate the effects of estrogens on reducing apoptosis of osteoblasts [85]. Estrogens can also modulate Wnt signaling in osteoblasts by regulating the levels of sclerostin, an inhibitor of Wnt signaling. Clinical studies have shown that the treatment of postmenopausal women with selective estrogen receptor modulators, such as raloxifene, leads to decreased sclerostin levels in patients [73]. Vica versa, inhibition of sclerostin by a humanized monoclonal antibody called romosozumab could enhance bone formation and stop bone loss in a study of postmenopausal women, similar to the effects of estrogen therapy [73].

On the cellular level, testosterone stimulates the development of osteoblasts via the enhancement of IL-1β signaling in precursor cells [73]. While estrogen is the key hormonal regulator of bone metabolism not only in women but also in men, Leydig cells can also regulate bone homeostasis besides testosterone secretion. Leydig cells are able to produce insulin-like 3 factor (Insl3), which stimulates osteoblast function [86]. Furthermore, Leydig cells can contribute to the synthesis of active vitamin D_3_ by enhancing its 25-hydroxylation [86]. As a consequence, male hypogonadism is associated with low levels of Insl3 and an increased risk of osteoporosis in patients [86].

### 4.2. Role of Steroid Hormones on Osteocytes

Osteocytes are long-lived cells derived from osteoblasts that are embedded in the bone matrix and can regulate bone remodeling upon changes to the mechanical forces acting on bone [87]. Importantly, osteocytes reside within the bone tissue and account for the majority of all bone cells [88]. They have long been considered quiescent bystander cells compared to osteoblasts and osteoclasts, however, recent studies demonstrated that osteocytes play a central role in regulating the dynamic interactions between bone cells [89]. Besides osteoblasts, osteocytes are the other major cellular source of RANKL, a key growth factor for the development of osteoclasts [89]. Osteocytes are connected to osteoblasts and osteoclasts by an extensive canalicular network. Their projections within the canaliculi communicate via gap functions and enable osteocytes to respond to mechanical stimuli [87]. An increasing amount of evidence suggests that abnormal osteocyte function plays a critical role in the pathogenesis of bone diseases, such as osteoporosis [88,89].

#### Effects of Glucocorticoids and Sex Steroids on Osteocytes

Osteocytes are important cellular targets of steroid hormones. Steroids cause profound effects on osteocyte differentiation and function. Glucocorticoids and sexual steroids influence the development and lifespan of osteocytes. Manolagas and colleagues showed that oxidative stress and reactive oxygen species (ROS) inhibit osteoblastogenesis and decrease the survival of osteocytes [90,91,92,93]. Estrogens and androgens decrease the level of intracellular ROS, while supraphysiological levels of glucocorticoids increase it in osteoblasts and osteocytes [90,91,92,93]. The main effect of estrogens is to decrease the RANKL/OPG ratio, while an excess of glucocorticoids increases via osteoblasts and osteocytes. It is also observed that gonadal steroid deficiency in patients enhances oxidative stress [84]. As a consequence, antioxidant treatment can prevent bone loss associated with estrogen or androgen deficiency in experimental animals [84]. Therefore, the authors concluded that age-related changes in glandular steroidogenesis and local ROS production might contribute to the pathogenesis of bone diseases, such as osteoporosis.

### 4.3. Role of Steroid Hormones on Osteoclasts

Osteoclasts are multinuclear cells of hematopoietic origin that can resorb the bone [94]. They are rich in lysosomal enzymes, e.g., tartrate-resistant acid phosphatase (TRAP), which is a marker of osteoclasts [94]. A key factor regulating osteoclast development is the receptor activator of nuclear factor κB (RANK) ligand (RANKL) produced by stromal cells in the bone microenvironment, such as osteoblasts and osteocytes [95]. However, RANKL can also be expressed by a number of hematopoietic cells (e.g., T cells and B cells) as well as by certain tumor cells [95]. Osteoprotegerin (OPG), a decoy receptor for RANKL, is also secreted by osteoblast-like cells, and it blocks the interaction of RANKL with its receptor RANK, leading to the inhibition of osteoclastogenesis [94]. The RANKL/OPG ratio determines the physiological balance of bone formation and turnover, with a higher ratio promoting excessive bone loss [94]. Besides RANKL, macrophage colony-stimulating factor (M-CSF) is also a critical factor for osteoclast precursor cell survival and differentiation [94]. During the process of bone remodeling, osteoclasts develop a tight connection with the bone surface by forming an actin ring structure, and then both the organic and inorganic matrix components break down due to the activity of osteoclasts by the simultaneous release of hydrochlorous acid and digestive enzymes, e.g., cathepsin K and matrix metalloproteases, into the resorption pit [94]. Osteoclasts are terminally differentiated cells, and osteoclast apoptosis is an important determinant of osteoclast activity [94].

#### Effects of Glucocorticoids and Sex Steroids on Osteoclasts

Glucocorticoids and sexual steroid hormones have significant effects on osteoclasts. Glucocorticoids influence the development and survival of osteoclasts mainly indirectly via the osteoblasts by modulating the expression of RANKL/OPG [84]. Osteoblastic expression of RANKL is increased, while OPG secretion by osteoblasts is decreased by glucocorticoids. In line with this, glucocorticoids stimulate the expression of collagenase 3 by posttranscriptional mechanisms to increase osteoclastic bone resorption [84]. Sex steroids exert their profound effects on bone metabolism via genomic and nongenomic effects. For estrogens, both direct and indirect effects on osteoclasts were described. Estrogens can suppress RANKL expression by osteoblasts, osteocytes, and T and B cells, as well as increase the production of the decoy receptor OPG. Estrogens were also found to be able to modulate the levels of osteoclastogenic cytokines (e.g., IL-1, IL-6, TNF-α, and M-CSF) [95,96,97,98,99,100,101,102]. In addition, recent studies also demonstrated the presence of estrogen receptors in osteoclasts [103,104]. Estrogens can modulate immune function and the activation of T cells [84]. Activated T lymphocytes produce osteoclastogenic factors, e.g., tumor necrosis factor α (TNFα) [84]. TNFα is a cytokine that stimulates stromal cell production of RANK ligand and M-CSF, this way enhancing osteoclastogenesis [84]. Estrogen is able to modulate T cell activation and suppress bone resorptive activity of osteoclasts via TNFα downregulation [84]. On the other hand, the lack of estrogens promotes RANKL expression on osteoblasts and osteocytes, leading to enhanced bone resorption. This excessive bone loss in women with postmenopausal osteoporosis can be reversed via treatment with denosumab, a human monoclonal antibody against RANKL [105]. Finally, locally produced steroid hormones could also exert an effect on bone cells, according to the latest data. Details of this pathway will be discussed next.

### 4.4. Role of Extraglandular Steroidogenesis in the Bone Microenvironment

Recent evidence indicated that osteoblasts not only respond to steroid hormones but can also synthetize testosterone from DHEA under pathological conditions during the development of prostate cancer bone metastases [106]. Intratumoral production of the adrenal androgen precursor DHEA allowed bone-forming osteoblasts to convert and secrete androgens to drive the development of osteosclerotic skeletal lesions [106]. Both human and murine osteoblasts were found to express Hsd3b2 and Cyp17a1 enzymes, suggesting that osteoblasts are capable of generating testosterone from DHEA [106]. In another publication authors also suggested a role for osteoblasts in promoting castration-resistant prostate cancer development by altering intratumoral steroidogenesis [107].

Similarly, our group recently identified a key role for extranglandular de novo steroidogenesis in osteolytic skeletal lesion formation by breast cancer and melanoma cells using RNA sequencing [108]. E0771/Bone and B16F10 tumor cells that expressed Cyp11a1 were capable of forming bone metastases in mice [108]. Furthermore, pregnenolone, the product of Cyp11a1 activity, was detected in high concentrations in the supernatants of different human and mouse osteotropic cancer cell lines by liquid chromatography/tandem mass spectrometry (LC-MS/MS) and ELISA [108]. The genetic deletion of Cyp11a1 by CRISPR/Cas9 mutagenesis in tumor cells or pharmacological inhibition of Cyp11a1 using aminoglutethimide protected animals from skeletal lesion formation and tumor-induced osteolysis in vivo [108]. Importantly, cancer cell-derived pregnenolone was able to drive the development of bone-resorbing osteoclasts by inducing the fusion of osteoclast precursors in an in vitro Cre-lox system-based novel fusion assay [108]. This effect of pregnenolone was mediated by a molecule called P4hb, which was able to promote the migration and fusion of osteoclast precursors [108]. Further, higher expression of Cyp11a1 in primary tumors was associated with a worse prognosis in patients with breast carcinoma based on in silico data [108].

**Figure 3:**
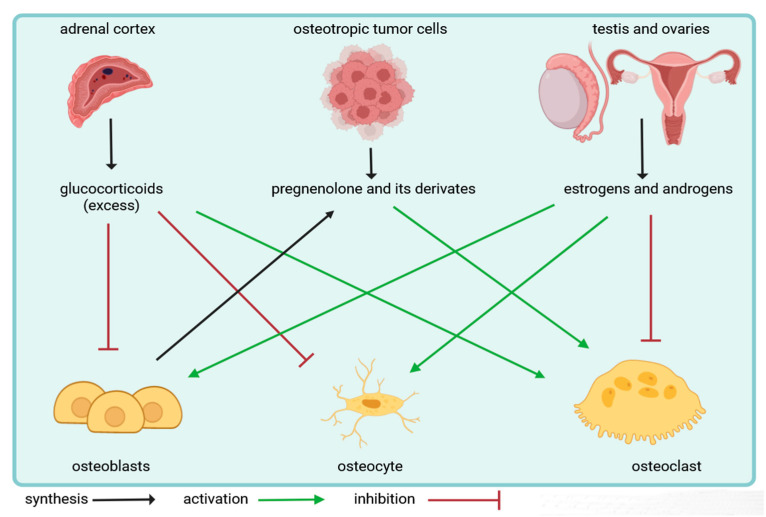
Overview of the local effects of steroid hormones on bone cells.

Finally, in another recent paper, the authors demonstrated that intratumoral androgen synthesis drives prostate cancer progression in patients as well [109]. They showed that Semaphorin 3C, a signaling molecule secreted by prostate cancer cells, can alter the expression of key steroidogenic enzymes, e.g., Cyp11a1 [109]. Moreover, Semaphorin 3C not only promotes androgen synthesis from cholesterol de novo but also downregulates enzymes involved in the androgen inactivation pathway [109]. The ability of semaphorin 3C to promote intratumoral androgen synthesis was confirmed by the authors in castration-resistant prostate cancer patients by conferring continued growth of prostate tumors under androgen deprivation therapy associated with Semaphorin 3C upregulation [109]. Other papers also implicated a role for insulin and Igf2 in de novo steroidogenesis in prostate cancer cells [110,111]. These are the first pieces of evidence for the unique role of extraglandular steroidogenesis occurring within the bone microenvironment during tumor development. However, future studies are required to better understand the role of local steroid production in the skeletal system under physiological and pathological conditions. Figure 3 summarizes the effects of glandular and extraglandular steroidogenesis on bone cells.

### 4.5. Role of Secosteroids in the Modulation of Bone Homeostasis

Besides being a lipid-soluble vitamin, vitamin D is also a steroid hormone. Precursors of active vitamin D are either produced in the skin from 7-dehydrocholesterol upon exposure to UVB light or obtained from plants and animal food as ergocalciferol (vitamin D_2_) or cholecalciferol (vitamin D_3_) [112,113]. The latter is then transported to the liver, where the first hydroxylation occurs and results in the generation of 25-hydroxyvitamin D_3_. Finally, vitamin D_3_ is activated by a second hydroxylation step in the kidneys. Although the primary function of vitamin D_3_ is the regulation of calcium metabolism, it also plays a key role in the regulation of bone homeostasis [114]. Importantly, it controls both bone formation and resorption by promoting osteoblast development and regulating the expression of RANKL on osteoblasts and osteocytes [115,116].

Recently, a surprising role of Cyp11a1 has been discovered in the metabolism of 7-dehydrocholestrol, the precursor of active vitamin D_3_. Namely, Cyp11a1 enzyme activity could lead to the generation of vitamin D_3_ hydroxyderivatives (so-called secosteroids) [117]. Although Cyp11a1-derived hydroxyderivatives of vitamin D_3_ are present in human serum and detected in the epidermis, we have just started to understand the biological role of this pathway [117]. Recently, Postlethwaite and colleagues reported that Cyp11a1-derived 20-hydroxyvitamin D_3_ can decrease joint damage in a mouse model of rheumatoid arthritis by changing inflammatory cytokine levels and altering lymphocyte subpopulations in the peripheral blood of the animals [118]. The authors propose 20-hydroxyvitamin D_3_ for the treatment of rheumatoid arthritis (RA) and other inflammatory joint diseases [118]; however, further experiments are required to better understand the role of secosteroids in the development of bone diseases.

## 5. Clinical Relevance

Synthetic steroids are extensively used in clinical practice as anti-inflammatory and immunosuppressive drugs for autoimmune diseases, organ transplantation, and in the treatment of leukemia. However, long-term use of these drugs can lead to serious side effects in the skeletal system. Patients with Cushing’s syndrome and steroid-induced osteoporosis develop excessive bone loss after a few months, while longer synthetic steroid treatment increases the risk of fractures of the spine and hip. Histomorphometric analysis of bone biopsies from patients receiving exogenous glucocorticoids revealed increased bone resorption and decreased bone formation at the cellular level. Glucocorticoid excess suppresses bone formation by inhibiting osteoblast differentiation and by promoting the apoptosis of osteoblasts and osteocytes. Excess glucocorticoids also increase bone resorption by enhancing osteoclast differentiation via increasing M-CSF and RANKL production and decreasing OPG secretion by osteoblasts and osteocytes [119].

Hypogonadism in adults is associated with excessive bone loss as well. In women, postmenopausal osteoporosis is linked with estrogen deficiency. Estrogens promote the apoptosis of mature osteoclasts by inhibiting the activity of RANK, decreasing RANKL expression, and increasing OPG secretion by osteoblasts and osteocytes, as well as suppressing the production of IL-1, IL-6, and TNFα in the skeletal system. Menopause-related estrogen deficiency accelerates both bone formation and resorption, but bone resorption surpasses the formation of bone, which results in a decrease in bone mineral density [120]. Finally, vitamin D_3_ deficiency can cause bone loss and fractures as well. Severe vitamin D_3_ deficiency leads to rickets in children and osteomalacia in adults. Glucocorticoid excess and hypogonadism are known to cause vitamin D_3_ deficiency [86,119].

Anti-resorptive agents (e.g., bisphosphonates, denosumab, estrogens, and selective estrogen receptor modulators (SERMs)) and bone anabolic agents (e.g., teriparatide) increase bone mineral density (BMD) and reduce the risk of fractures in patients. Bisphosphonates can inhibit osteoclast activation and prevent increased bone resorption associated with excessive bone loss. Denosumab, a human monoclonal antibody against RANKL, can reverse bone loss associated with synthetic steroid treatment. SERMs (e.g., raloxifene) have estrogen activity and prevent bone loss as well as improve BMD. Anabolic agents, e.g., PTH analog teriparatide, can stimulate bone formation and increase peak bone mass in patients with osteoporosis [119,120]. However, the pathogenesis of steroid-associated bone loss is still not completely understood; therefore, future research is required to provide insights into the molecular mechanisms of steroid hormone action at the cellular level.

## 6. Conclusions and Future Perspectives

Steroid hormones are key regulators of homeostasis and control endocrine functions in the body [1]. Steroid production is mainly studied in the adrenal zona glomerulosa, fasciculata and reticularis cells, testicular Leydig cells, ovarian granulosa, as well as theca interna and placental syncytiotrophoblast cells [2]. Besides the centrally regulated glandular steroidogenesis, local de novo steroid production also occurs in several other tissues [17,18]. Among these, pregnenolone and its derivates play an important role in the regulation of bone homeostasis and bone cells, i.e., osteoblasts, osteocytes, and osteoclasts respond to steroid hormones. Glucocorticoids and sexual steroids exert a direct effect on osteoblasts, while the actions of steroid hormones on osteoclasts are rather indirect and mediated via osteoblasts. Additionally, steroid hormones influence osteocytes as well. Estrogens and androgens stimulate the survival and development of both osteoblasts and osteocytes, while glucocorticoids inhibit them. Vica versa, glucocorticoids enhance osteoclast formation, while sexual steroids inhibit it by lowering the RANKL/OPG ratio. Recent evidence indicated that osteoblasts and osteoclasts can also respond to steroid hormones produced by extranglandular steroidogenesis within the bone microenvironment. We and others demonstrated that pregnenolone and its derivates secreted by osteotropic tumor cells and osteoblasts are able to stimulate androgen production and osteoclast development and function [106,107,108,109,110,111]. Therefore, understanding the cellular and molecular effects of steroid hormones in the skeletal system is expected to facilitate the development of new therapies for the treatment of bone disease.

Novel discoveries and innovations in the area of steroid hormone research are promoted by technological and methodological advancements in the field. To identify locally produced steroid hormones and novel steroid synthesis pathways in different tissues, profiling, and quantification of all steroid hormones and their metabolites by liquid chromatography/tandem mass spectrometry is now widely available. Further, combining mass spectrometry with ChIP sequencing to selectively isolate chromatin-associated proteins may facilitate the understanding of the role of receptors of steroid hormones, which exert their effect by regulating the gene expression in target tissues [18]. Steroidogenic enzyme activity in living cells can be measured by chemoproteomics-based protein profiling techniques [121]. Finally, recently developed transgenic mice could also be useful tools to study the effect of steroid hormones in vivo, e.g., the Cyp11a1-H2b-mCherry reporter line [18] and the Cyp11a1-Gfp-Cre animals [122].

Last but not least, synthetic steroids are extensively used in clinical practice, and long-term use of these drugs can lead to serious side effects in the skeletal system. A better understanding of local steroidogenesis within the skeletal system and steroid hormone-mediated regulation of bone cells may lead to the discovery of novel therapeutic strategies to eliminate undesirable side effects of synthetic steroids, ensuring the maintenance of physiological bone homeostasis.
